# Multi-Domain Variational Autoencoders for Combined Modeling of MRI-Based Biventricular Anatomy and ECG-Based Cardiac Electrophysiology

**DOI:** 10.3389/fphys.2022.886723

**Published:** 2022-06-08

**Authors:** Marcel Beetz, Abhirup Banerjee, Vicente Grau

**Affiliations:** ^1^ Department of Engineering Science, Institute of Biomedical Engineering (IBME), University of Oxford, Oxford, United Kingdom; ^2^ Radcliffe Department of Medicine, Division of Cardiovascular Medicine, University of Oxford, Oxford, United Kingdom

**Keywords:** multi-domain variational autoencoder, combined electrocardiogram and cardiac anatomy generation, cardiac disease classification, point clouds, cine magnetic resonance imaging, cardiac electrophysiology, time series analysis, geometric deep learning

## Abstract

Human cardiac function is characterized by a complex interplay of mechanical deformation and electrophysiological conduction. Similar to the underlying cardiac anatomy, these interconnected physiological patterns vary considerably across the human population with important implications for the effectiveness of clinical decision-making and the accuracy of computerized heart models. While many previous works have investigated this variability separately for either cardiac anatomy or physiology, this work aims to combine both aspects in a single data-driven approach and capture their intricate interdependencies in a multi-domain setting. To this end, we propose a novel multi-domain Variational Autoencoder (VAE) network to capture combined Electrocardiogram (ECG) and Magnetic Resonance Imaging (MRI)-based 3D anatomy information in a single model. Each VAE branch is specifically designed to address the particular challenges of the respective input domain, enabling efficient encoding, reconstruction, and synthesis of multi-domain cardiac signals. Our method achieves high reconstruction accuracy on a United Kingdom Biobank dataset, with Chamfer Distances between reconstructed and input anatomies below the underlying image resolution and ECG reconstructions outperforming multiple single-domain benchmarks by a considerable margin. The proposed VAE is capable of generating realistic virtual populations of arbitrary size with good alignment in clinical metrics between the synthesized and gold standard anatomies and Maximum Mean Discrepancy (MMD) scores of generated ECGs below those of comparable single-domain approaches. Furthermore, we observe the latent space of our VAE to be highly interpretable with separate components encoding different aspects of anatomical and ECG variability. Finally, we demonstrate that the combined anatomy and ECG representation improves the performance in a cardiac disease classification task by 3.9% in terms of Area Under the Receiver Operating Characteristic (AUROC) curve over the best corresponding single-domain modeling approach.

## 1 Introduction

Healthy cardiac function of the human heart consists of complex interactions between anatomical deformations and electrophysiological conduction patterns which vary considerably between individuals in the population. Accounting for this variability is of high importance in clinical practice as it heavily influences the accuracy of cardiovascular disease diagnosis and treatment. Consequently, it is also a core objective of computational modeling approaches of cardiac anatomy and function to correctly represent these inter-person differences and enable more personalized and accurate computer models. Two of the most commonly used modalities in clinical practice to assess healthy cardiac function on both an individual and a population level are, respectively, the cardiac Magnetic Resonance Imaging (MRI) (Stokes and Roberts-Thomson, 2017) and the Electrocardiogram (ECG) (Macfarlane and Lawrie, 2010).

Due to its high soft-tissue contrast and lack of ionizing radiation combined with high temporal resolution, cardiac cine MRI is currently considered the gold-standard for image-based cardiac function analysis (Stokes and Roberts-Thomson, 2017). It has also been extensively used to determine normal cardiac behavior and investigate inter-patient differences. To this end, several image-based atlases of the heart with associated statistical shape models of cardiac anatomy and function have been developed for a variety of different populations and cardiac substructures (Bai et al., 2015; Nagel et al., 2021). In these approaches, a mean template shape is typically created from a distribution of image-derived cardiac shapes, followed by Principal Component Analysis (PCA) to model population variability (Tavakoli and Amini, 2013; Bai et al., 2015; Piazzese et al., 2017). More recently, deep learning approaches based on Variational Autoencoders (VAE) or Generative Adversarial Networks (GAN) have also been explored for this purpose (Litjens et al., 2017; Bello et al., 2019; Biffi et al., 2020; Gilbert et al., 2020; Beetz et al., 2021b; Rezaei, 2021). The resulting statistical models have a variety of use cases, including the prediction of certain cardiac disease events (Acero et al., 2022), the association analysis of cardiac shape and disease risk factors (Mauger et al., 2019), and the generation of virtual populations for physiological simulations (Mincholé et al., 2019; Niederer et al., 2020; Romero et al., 2021).

The ECG offers an easy and non-invasive procedure to capture and visualize the electrical conduction patterns of the heart and is therefore widely used in clinical diagnosis and electrophysiology modeling (Macfarlane and Lawrie, 2010). Similar to cine MRI, considerable research has been focused on capturing population variability in the ECG signals. For example, PCA has been applied to ECG data to derive respiratory signals (Langley et al., 2009), estimate the effect of diabetes on ECG parameters (Kalpana et al., 2013), or classify ECG beats (Martis et al., 2013). GAN and VAE-based approaches have more recently been investigated for the task of virtual ECG generation and to analyze ECG shape variations across the population (Delaney et al., 2019; Zhu et al., 2019; Kuznetsov et al., 2021).

However, in all aforementioned works, inter-subject variability was modeled based on either MRI or ECG information separately in a single-domain setting. This neglects the complex, non-linear relationships between anatomical deformations and electrophysiological conduction, and therefore inhibits a more holistic understanding of cardiac function and its variability across the human population. Hence, the objective of this work is to combine both cine MRI-based cardiac anatomy information and ECG-based electrophysiology information across a whole population in a single data-driven modeling approach and study their variations and interactions in this multi-domain setting. To this end, we propose a multi-domain variational autoencoder framework consisting of multiple domain-specific branches and a latent space shared across all branches for cross-domain information exchange. The design of the individual branches, loss function, and training procedure are specifically tailored to a multi-domain dataset consisting of both MRI-based cardiac anatomy information and ECG-based electrophysiology signals. Anatomical information is represented as high-resolution and multi-class 3D point clouds reconstructed from cine MRI acquisitions and can be efficiently processed by the point cloud-based deep learning branches. Anatomies at both the End-Diastolic (ED) and End-Systolic (ES) phases of the cardiac cycle are used together with the corresponding ECG signals to give the network access to both spatial and temporal information.

Similar to the single-domain shape modeling approaches, the multi-domain VAE has a variety of possible use cases in both clinical and research settings, such as problem-specific dimensionality reduction of high-dimensional data, interpretable shape analysis of both spatial and temporal data, explainable cardiac disease identification and prediction, or the generation of virtual population cohorts for mechanical and electrophysiological computer simulations or to augment datasets for training machine learning or deep learning classifiers or regressors.

To the best of our knowledge, this is the first deep learning method to capture the combined cardiac anatomy and electrophysiology data in a single model. In summary, our contributions are as follows:

• We present a novel multi-domain variational autoencoder capable of modeling combined cardiac anatomy and ECG data.
• We provide a detailed explanation of the preprocessing steps, network architecture, loss function, and training procedure.
• We assess the VAE’s ability to reconstruct multi-domain data on a United Kingdom Biobank dataset (Petersen et al., 2015, 2013) of 1,000 cases and compare the reconstruction performance with multiple single-domain benchmarks.
• We evaluate the VAE’s capability to generate realistic virtual populations of combined anatomy and ECG data and perform a comparative analysis with the gold standard test set and multiple single-domain benchmarks.
• We investigate the VAE’s latent space with regards to its interpretability and degree of disentanglement.
• We develop and evaluate a machine learning classifier for cardiac disease prediction from the VAE’s latent space.
• We include a detailed discussion of our findings and a pertinent literature review.

A preliminary version of this work was presented in Beetz et al. (2022). This paper provides a more comprehensive explanation of the methodology, additional new experiments including comparisons with various benchmarks and application to 150 pathological cases, and a substantially expanded discussion and literature review.

## 2 Materials and Methods

In this section, we describe the multi-domain dataset used for method development (Section 2.1) and explain the required preprocessing steps (Section 2.2) as well as our method’s architecture (Section 2.3, Section 2.4, Section 2.5), loss function (Section 2.6), and training procedure (Section 2.7).

### 2.1 Dataset

We conduct our research work using 1,300 subjects from the United Kingdom Biobank imaging study (Petersen et al., 2013) for which paired cardiac cine Magnetic Resonance (MR) images and electrocardiograms were acquired (Petersen et al., 2015). All cine MR short-axis images had a voxel resolution of 1.8 × 1.8 × 8.0 mm^3^ and typical image dimensions of 208 × 168–210, while the cine MR long-axis images had a voxel resolution of 1.8 × 1.8 × 6.0 mm^3^ with typical image dimensions of 208 × 126–180 (Petersen et al., 2015). 1,150 subjects were assumed to be healthy individuals, while 150 cases suffered from at least one pathology related to the cardiovascular system. These cardiovascular disease cases were identified following the same procedure outlined in Bai et al. (2020), based on the self-reported disease codes in the United Kingdom Biobank (see Supplementary Table S1). We select 1,000 presumably healthy cases for the initial method development and the experiments in Section 3.2, Section 3.3, Section 3.4, and Section 3.5. The dataset is randomly split into training, validation, and test sets of sizes ∼800, ∼50, and ∼150, respectively, to give the network access to enough cases for training, while at the same time retaining a sufficiently high number of cases for method evaluation. We use the remaining 150 healthy and 150 diseased cases for our cardiac disease classification experiment described in Section 3.6.

### 2.2 Domain-Specific Data Preprocessing

**FIGURE 1:**
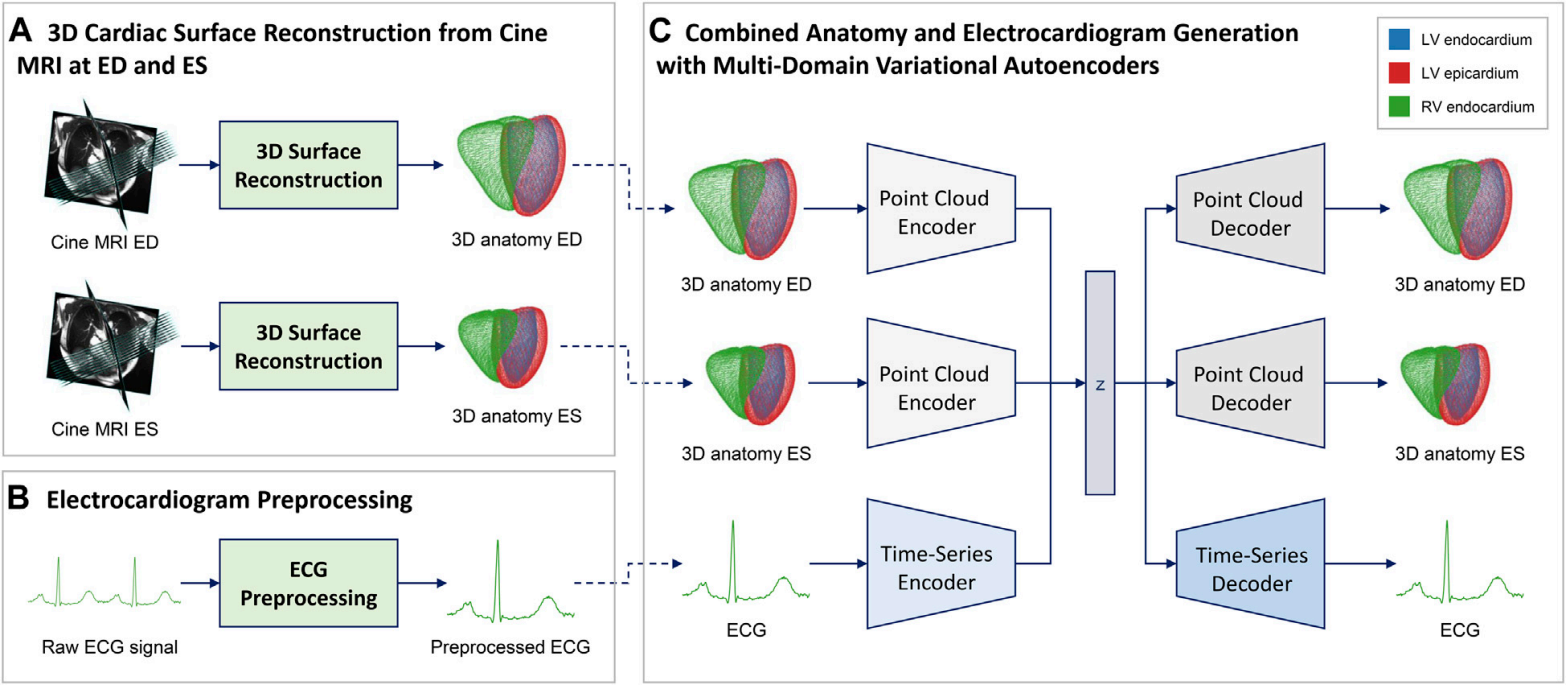
Overview of the proposed combined anatomy and ECG modeling pipeline. We first reconstruct point cloud representations of the 3D biventricular anatomy at the ED and ES phase of the cardiac cycle **(A)** and preprocess the raw ECG acquisitions **(B)** to create a multi-domain dataset. We then use this data to train a multi-domain variational autoencoder **(C)** to capture combined cardiac anatomy and electrophysiology information in a single model. The VAE architecture **(C)** consists of three separate encoder-decoder branches, one for each network input (ED anatomy, ES anatomy, ECG), that share a common latent space for cross-modal information exchange. Each branch architecture is specifically tailored to the requirements of the respective input type, i.e. point clouds for anatomy and time series for ECG processing (Figure 2).

In order to extract the anatomical and physiological information required for training our multi-domain VAE from the raw cine MRI and ECG signals, we first apply various preprocessing steps to the data from each modality (Figure 1A,B). Regarding the imaging data (Figure 1A), we first segment both short- and long-axis images of the cine MRI acquisition into four classes that delineate the anatomical substructures of interest (Left Ventricular (LV) cavity, LV myocardium, Right Ventricular (RV) cavity, and background) using the fully convolutional neural networks as detailed in Banerjee et al. (2021) and Bai et al. (2018). Next, we use the obtained segmentation masks from the short-axis images to identify the ED and ES phases of the cine MRI sequence for each case as anatomical representations of the extreme ends of the cardiac cycle (Banerjee et al., 2021). The final 3D point clouds of the biventricular anatomy are reconstructed at both ED and ES phases from the selected slices using the approach described in Beetz et al. (2021a). For ECG data (Figure 1B), the United Kingdom Biobank provides both a raw acquisition consisting of multiple heart beats, as well as a combined ECG signal that averages the information from multiple cardiac cycles into a single one-heartbeat representation for each lead. In this work, we focus on the lead II signals, since they provide a good view of the P and R waves, are predictive of many cardiac arrhythmias, and are also used by previous methods (Delaney et al., 2019; Wang et al., 2019). We choose the average lead II signal in each case as our ECG data and apply the standardization step, i.e. subtracting the mean value from each data instance and dividing by the standard deviation, to each resulting time series. The preprocessed ECG is then combined with the corresponding 3D point cloud reconstructions of the biventricular anatomy at the ED and ES phases of each case to form the multi-domain dataset used for method development.

### 2.3 Multi-Domain Variational Autoencoder

In order to capture the combined anatomy and ECG data obtained from the preprocessing steps, we propose a multi-domain *β*-VAE (Higgins et al., 2017) architecture with three branches that share a common latent space for inter-modal information sharing (Figure 1C).

Each of the three branches has an encoder-decoder structure and is responsible for processing one of the three inputs, namely the ED anatomy point cloud, the ES anatomy point cloud, and the ECG. The encoder outputs of the three branches are tasked with predicting the mean and standard deviation vectors of the multivariate Gaussian distribution of the latent space following the standard variational autoencoder setting (Kingma and Welling, 2013). A 12-dimensional vector is sampled from this distribution and passed into each decoder of the three branches which aim to reconstruct the input of their corresponding encoder branch. The reparameterization (Kingma and Welling, 2013) trick is applied during training. The architectures of each of the three branches are specifically designed to enable efficient processing of the respective data type (i.e. point clouds and time series) and are described in greater detail in Section 2.4 and Section 2.5. The two anatomy branches share the same network architecture (Section 2.4) but maintain separate trainable network parameters, while the ECG branch exhibits a different design (Section 2.5).

### 2.4 Point Cloud Branches

**FIGURE 2:**
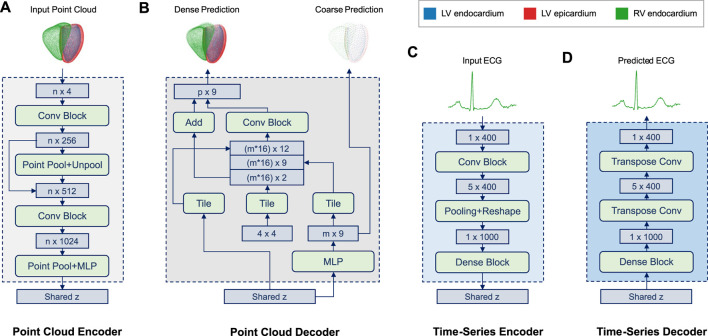
Overview of the encoder and decoder architectures of both the point cloud **(A,B)** and time series **(C,D)** branches of the multi-domain VAE. The input point cloud **(A)** encodes the biventricular anatomy as a set (n = 36,000) of 4D vectors (x,y,z coordinates and the class label of each point), while a separate set of 3D point coordinates is used for each of the three cardiac substructures in the output point cloud **(B)**. The point cloud decoder **(B)** outputs both a coarse, low-dimensional (2,250 points) and a dense, high-dimensional (36,000 points) representation of the cardiac anatomy. The former represents the final output used for further processing, while the latter primarily facilitates the training process in this work (Eq. 5) by first focusing on an approximate reconstruction and later putting more emphasis on the dense output. Both input and output ECG time series **(C,D)** are represented as 400-dimensional vectors. Both encoders **(A,C)** are tasked with predicting the shared latent space *z*. The time-series decoder **(D)** follows a symmetric design to its encoder **(C)** and aims to reconstruct the input ECG signal from the latent space during training. (Background colors of architecture blocks are consistent with Figure 1)

The architecture of the two anatomy branches of the multi-domain VAE (Figure 2A,B) follows an extended version of the point completion network (Yuan et al., 2018) and its adaptations to cardiac image analysis (Beetz et al., 2021b).

The network input point clouds are encoded as sets of 36,000 4-dimensional vectors consisting of the 3D coordinates and the class label of each point which indicates its cardiac substructure (LV endocardium, LV epicardium, RV endocardium). Point clouds are then passed through the encoder (Figure 2A), which resembles a multi-class extension of the Pointnet (Qi et al., 2017a) architecture. Similar to (Qi et al., 2017b), it consists of two stacked Pointnets that are connected via a skip connection as well as a pooling and an unpooling step. Furthermore, we add additional fully connected layers before the latent space to enable easier information sharing. The encoder outputs are then concatenated with the respective outputs of the other two branches before the variational sampling step is applied. The sampled latent space vector is then provided as input into the decoder (Figure 2B) where a multi-layer perceptron (MLP) is first tasked with creating a low-resolution multi-class point cloud with 2,250 points. This coarse point cloud aims to represent the biventricular anatomy on a global level and is primarily used to stabilize the training process of the network in the early stages. The second step of the decoder follows the design of FoldingNet (Yang et al., 2017) and processes the previous low-resolution output, the sampled latent space vector, and a set of tiled point grids to generate a high-resolution multi-class point cloud with 36,000 points as the final network output. For both the low and high resolution output point clouds, each class is represented by a separate set of 750 and 12,000 3D coordinates, respectively.

### 2.5 Time-Series Branches

The architecture of the ECG branch combines convolutional, pooling, and dense layers to capture both local and global patterns at different scales (Figure 2C,D). The encoder (Figure 2C) receives each ECG time series as a 400-dimensional input vector and passes it through two convolutional blocks, each of which consists of a 2D convolution, an Exponential Linear Unit (ELU) activation function, and a batch normalization layer. This is followed by an average pooling layer and two fully connected layers, which output the mean and standard deviation vectors of the multivariate normal distribution of the latent space, respectively. Next, the sampled vector *z* from the shared latent space distribution of the multi-branch autoencoder is fed through a dense block with two fully connected layers at the beginning of the decoder (Figure 2D). Subsequently, two transposed 2D convolutions are applied to obtain the 400-dimensional ECG time series reconstruction as the final network output.

### 2.6 Loss Function

Following the formulation of the *β*-VAE (Higgins et al., 2017) framework, our loss function *L*_*total*_ is composed of the sum of a reconstruction loss term *L*_*recon*_ and a regularizing term *L*_*KL*_ weighted by the parameter *β*, as

$$L_{\mathrm{total}}=L_{\mathrm{recon}}+\beta *L_{KL}.$$ (1)

We use the Kullback-Leibler divergence between the latent space distribution *Q* (*z*|*X*) and the multivariate standard Gaussian prior distribution *P*(*z*) as the regularizing loss term *L*_*KL*_, where *X* refers to the VAE inputs and *z* to the VAE's latent space. This encourages each latent space component to follow a normal distribution with zero mean and standard deviation of one, which we choose as our prior *P*(*z*).

$$L_{KL}=D_{KL}\left[Q\left(z|X\right)\|P\left(z\right)\right]$$ (2)

The reconstruction loss *L*_*recon*_ consists of three loss terms, one for each of the three branches in the multi-domain autoencoder. It incentivizes the VAE to output anatomy and ECG signals that are as close as possible to the respective inputs, which we consider to be our physiologically accurate gold standard for network training.

$$L_{\mathrm{recon}}=L_{ED}+L_{ES}+\gamma *L_{\mathrm{ECG}}$$ (3)

We introduce a parameter *γ* to control the importance of the ECG reconstruction during training.

We choose the mean squared error between the reconstructed ECG signals *x*_*n*_ and the gold standard ECG signals *y*_*n*_ across *N* time steps as our ECG loss term *L*_*ECG*_ to put more emphasis on correctly capturing less common values, such as the R-peak of the ECG signal.

$$L_{\mathrm{ECG}}=\frac{1}{N}\overset{N}{\underset{n=1}{\sum }}{\left(x_{n}-y_{n}\right)}^{2}$$ (4)

Each of the two anatomy loss terms *L*_*ED*_ and *L*_*ES*_ consists of the weighted sum of a coarse and a dense loss term over all three classes *C* corresponding to the respective cardiac substructures. We consider each part of the anatomy as equally important in the loss function and therefore do not use any class-specific weighting parameter.

$$L_{ED/ES}=\overset{C}{\underset{i=1}{\sum }}\left(L_{\mathrm{coarse,i}}+\alpha *L_{\mathrm{dense,i}}\right)$$ (5)

The coarse loss term measures the difference between the low-density output of the point cloud decoder and the ground truth, while the dense loss term compares the high-density output point cloud with the same ground truth. The weighting parameter *α* is used during training to first prioritize a good global structure of the coarse prediction and then gradually put increasing emphasis on local accuracy in the dense point cloud prediction.

Both the coarse and dense loss terms are calculated using the Chamfer Distance (CD) between the point cloud predicted by the network *P*_1_ and the ground truth input point cloud *P*_2_.

$$CD\left(P_{1},P_{2}\right)=\frac{1}{2}\left(\frac{1}{\left|P_{1}\right|}\underset{x\in P_{1}}{\sum }\underset{y\in P_{2}}{\min}\|x-y{\|}_{2}+\frac{1}{\left|P_{2}\right|}\underset{y\in P_{2}}{\sum }\underset{x\in P_{1}}{\min}\|y-x{\|}_{2}\right)$$ (6)

Since the Chamfer Distance aims to find the closest point in the ground truth point cloud for each point in the input point cloud and vice versa, it can be considered as an approximate surface-to-surface distance on point cloud data between the respective anatomical shapes.

### 2.7 Implementation and Training

Our deep learning experiments are conducted on a GeForce RTX 2070 Graphics Card with 8 GB memory. We use TensorFlow (Abadi et al., 2016) and Scikit-learn (Pedregosa et al., 2011) for our deep learning and machine learning implementations, respectively. All VAEs are trained using the Adam optimizer (Kingma and Ba, 2014) with a mini-batch size of 4, which we empirically found to provide a good balance between the memory and time constraints of our setup and the improved gradient quality during network training. The training duration is set to 150,000 steps based on the convergence of the loss function in the validation dataset. We set all loss weighting parameters to small values (*α* and *β* to 0.01, *γ* to 0.1) at the start of training to focus on obtaining a good coarse reconstruction of the two anatomy point clouds. We then gradually increase both *α* and *γ* to improve local prediction quality in both anatomy and ECG outputs. After both parameters have reached a value of 1, we increase the *β* parameter using a variation of the monotonic annealing schedule (Bowman et al., 2015) to improve the latent space quality. We stop the *β* value at 0.25, which we have empirically found to provide a good balance between overall reconstruction quality and latent space quality.

## 3 Experiments and Results

We evaluate the proposed multi-domain VAE in terms of its performance in multiple tasks. First, we investigate its ability to correctly reconstruct paired input data from all three domains (Section 3.2). Second, we assess its ability to generate virtual populations of realistic ECGs and anatomy point clouds, both within and across the different domains (Section 3.3, Section 3.4). Third, we analyse the effect of certain latent space changes on the reconstructed ECG and anatomy shapes to gain a better understanding of the latent space distribution (Section 3.5). Finally, we compare the compressed latent space representation of the proposed multi-domain VAE with its single-domain counterparts in a cardiovascular disease classification task (Section 3.6). We propose multiple different metrics for the outlined experiments to account for the different data types and objectives (Section 3.1).

### 3.1 Evaluation Metrics

In order to assess the VAE’s ECG reconstruction quality, we follow the metrics suggested by Zhu et al. (2019), which allows us to compare our results with the task of ECG-only generation without any image-based anatomy information. Accordingly, we use the Root Mean Squared Error (RMSE) as our first metric to quantify the distance between predicted ECG time series *x* and ground truth ECG time series *y* in our test dataset, each with a length of *N* time steps.

$$RMSE=\sqrt{\frac{1}{N}\overset{N}{\underset{n=1}{\sum }}{\left(x_{n}-y_{n}\right)}^{2}}$$ (7)

In addition, our second ECG reconstruction metric, Percentage Root Mean Squared Distance (PRD), provides a relative and normalized quantification of the reconstruction performance.

$$PRD=\sqrt{\frac{1}{\sum_{n=1}^{N}x_{n}^{2}}\overset{N}{\underset{n=1}{\sum }}{\left(x_{n}-y_{n}\right)}^{2}}*100$$ (8)

The anatomy reconstruction quality achieved by our VAE is evaluated using the average Chamfer Distance (Eq. 6) between the predicted and ground truth point clouds of the test dataset for both the ED and ES phases.

Similar to work by Delaney et al. (2019) on ECG-only generation, we propose the Maximum Mean Discrepancy (MMD) (Gretton et al., 2012) between two randomly generated distributions as a metric to assess the generative ability of our network. Hereby, *K* refers to the Gaussian kernel and *x* and *y* refer to the two sample distributions of sequences with sizes *n* and *m* respectively.

$$MMD={\left[\frac{1}{n\left(n-1\right)}\overset{n}{\underset{i=1}{\sum }}\overset{n}{\underset{j\neq i}{\sum }}K\left(x_{i},x_{j}\right)+\frac{1}{m\left(m-1\right)}\overset{m}{\underset{i=1}{\sum }}\overset{m}{\underset{j\neq i}{\sum }}K\left(y_{i},y_{j}\right)-\frac{2}{nm}\overset{n}{\underset{i=1}{\sum }}\overset{m}{\underset{j=1}{\sum }}K\left(x_{i},y_{j}\right)\right]}^{\frac{1}{2}}$$ (9)

In order to evaluate the quality of the generated anatomies at ED and ES separately, we select the widely used clinical evaluation metrics LV volume, RV volume, and myocardial mass. In addition, we choose the Stroke Volume (SV) (Eq. 10) and Ejection Fraction (EF) (Eq. 11) metrics for both the LV and RV, to assess the correspondence between the generated anatomies at ED and ES.

SV=EDV−ESV. (10)

$$EF=\frac{SV}{EDV}\times 100.$$ (11)

Here, EDV and ESV refer to ED volume and ES volume, respectively. Furthermore, we select the Area Under the Receiver Operating Characteristic (AUROC) curve to evaluate the performance in the binary cardiac disease classification task.

### 3.2 Reconstruction Ability

**FIGURE 3:**
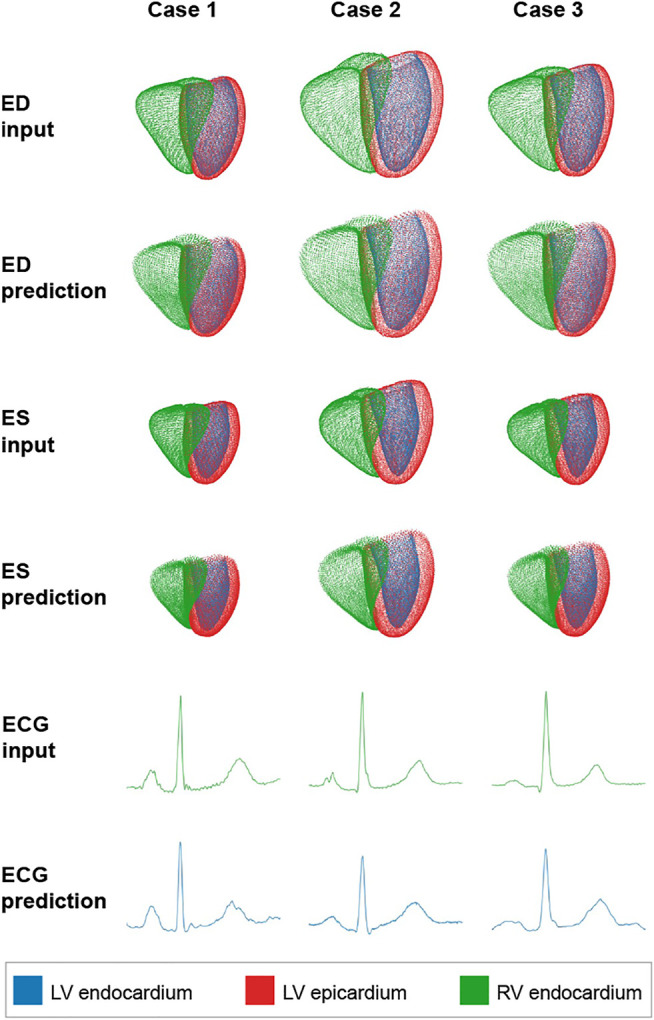
Qualitative reconstruction results of the proposed method for three sample cases.

We first focus on the network’s ability to accurately reconstruct both the two input point clouds and the input electrocardiogram. To this end, we pass the ED point cloud, the ES point cloud, and the ECG time series of each case of the test dataset through the network and compare the network’s predicted outputs with the respective inputs. Figure 3 shows input and prediction data of three such sample cases.

We observe good global and local alignment between inputs and predictions of both point cloud and time series data. Class information in the form of three anatomical substructures is also accurately reconstructed for both ED and ES point clouds. Next, we quantify our method’s reconstruction ability on the test dataset using separate metrics for each modality.

For the ECG data, we select the RMSE (Eq. 7) and PRD (Eq. 8) metrics to determine our method’s reconstruction error. We apply min-max normalization to both the input and predicted time series data before calculating the metrics, in order to compare the obtained values with the performance of multiple approaches proposed by Zhu et al. (2019) for single-domain ECG-only generation using the MIT-BIH dataset (Goldberger et al., 2000) (Table 1). In addition, we train and evaluate a separate VAE on only the ECG signals of our United Kingdom Biobank dataset as a benchmark method for our multi-domain VAE. It follows the encoder and decoder architecture presented in Figure 2C,D and uses the same ECG data and preprocessing steps as our proposed approach, allowing for a direct comparison (Table 1).

**TABLE 1:** ECG reconstruction results of multiple methods on different datasets.

Method	Dataset	RMSE	PRD
BiLSTM-CNN GAN *	MIT-BIH	0.22	51.80
BiLSTM-GRU *	MIT-BIH	0.31	74.05
BiLSTM-LSTM *	MIT-BIH	0.35	84.80
BiLSTM-MLP *	MIT-BIH	0.61	147.73
ECG VAE	United Kingdom Biobank	0.16	26.51
Multi-Domain VAE (Proposed)	United Kingdom Biobank	0.17	27.45

*Values obtained directly from Zhu et al. (2019)

We find that the proposed multi-domain VAE method achieves lower reconstruction errors than the ones reported by Zhu et al. (2019) for any of their architectures, both in terms of RMSE and PRD, despite the more challenging task of combined anatomy and electrocardiogram generation. However, this result should only be interpreted as an approximate marker of the reconstruction quality of our method instead of a direct outperformance, since different datasets and signal preprocessing steps were used in each analysis. For example, while the proposed approach uses the ECG signals averaged to one cardiac cycle from the United Kingdom Biobank, Zhu et al. (2019) did not mention the usage of averaged signals. Compared to the VAE trained on only ECG signals on the same United Kingdom Biobank dataset, our method achieves similar results for both evaluation metrics.

We quantify the reconstruction ability of our method for the point cloud data of the test dataset using the Chamfer Distance (Eq. 6). The resulting values for both ED and ES reconstructions, split by the three cardiac substructures, are reported in Table 2.

**TABLE 2:** ED and ES anatomy reconstruction results of our method on the test dataset.

Phase	Class	Chamfer Distance (mm)
ED	LV endocardium	1.37 (±0.40)
	LV epicardium	1.29 (±0.29)
	RV endocardium	1.42 (±0.29)
ES	LV endocardium	1.11 (±0.39)
	LV epicardium	1.23 (±0.45)
	RV endocardium	1.35 (±0.55)

Values represent mean (±standard deviation)

We find low distance values that are smaller than the voxel sizes of the image acquisitions (1.8 × 1.8 × 8.0 mm^3^) used to generate the 3D point clouds for both the ED and ES phases as well as for all cardiac substructures. Distances are slightly larger for the right ventricle compared to left ventricular structures and for the ED phase than for the ES phase.

### 3.3 Generative Ability

**FIGURE 4:**
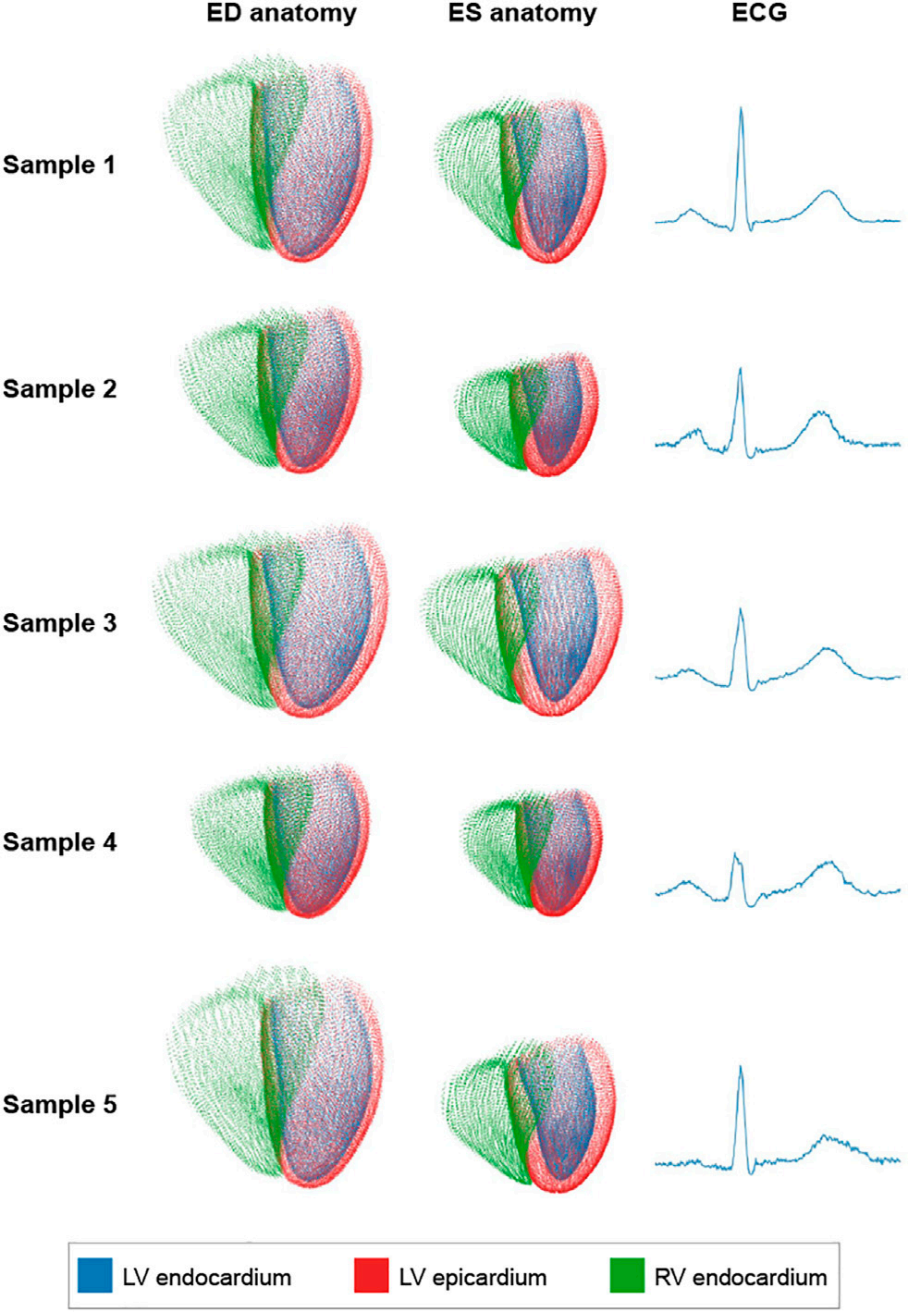
Five randomly generated sample outputs where each row presents one case.

In order to assess our network’s ability to generate diverse populations with realistic anatomies and ECGs, we randomly sample from the latent space distribution and pass the resulting vectors through the three branches of the decoder. The mean and standard deviation values of the multivariate normal distribution of the latent space are determined based on the averaged encoder outputs of the training data. Hence, every component of the latent space is involved in the sampling step. Five randomly generated decoder outputs, each consisting of an ED anatomy point cloud, an ES anatomy point cloud, and an ECG, are depicted in Figure 4.

We observe that all outputs follow realistic shapes and sizes while maintaining a good amount of diversity between different cases. For example, the case in the first row exhibits considerably larger heart sizes at both ED and ES and a noticeably higher R-peak in the electrocardiogram as compared to the case in the fourth row.

Next, we evaluate the multi-domain VAE’s capability for ECG generation on a population level. To this end, we synthesize 500 virtual electrocardiogram signals from randomly sampled latent space vectors and calculate their MMD (Eq. 9) with respect to the ECGs in our test dataset. We repeat the same procedure for the ECG-only VAE to enable a comparison of our multi-domain approach with a single-domain method on the same dataset. We also randomly split the test dataset into two subsets and determine the MMD between these two subsets to obtain a gold standard benchmark for desired ECG population similarity. The resulting values are reported in Table 3, together with MMD scores obtained from different approaches by Delaney et al. (2019) on the MIT-BIH dataset (Goldberger et al., 2000) and by Kuznetsov et al. (2021) on the LUDB dataset (Kalyakulina et al., 2020) for ECG-only generation.

**TABLE 3:** ECG generation results of multiple methods based on different datasets.

Method	Dataset	MMD
4CNN GAN (Delaney et al., 2019)*	MIT-BIH	1.03 × 10^–3^
4CNN BiLSTM GAN (Delaney et al., 2019)*	MIT-BIH	1.13 × 10^–3^
VAE (Kuznetsov et al., 2021)*	LUDB	3.83 × 10^–3^
Gold standard (test dataset)	United Kingdom Biobank	1.40 × 10^–4^
ECG VAE	United Kingdom Biobank	3.05 × 10^–5^
Multi-Domain VAE (Proposed)	United Kingdom Biobank	3.54 × 10^–5^

*Values obtained directly from Delaney et al. (2019) or Kuznetsov et al. (2021)

Our method achieves lower MMD scores than all other methods by a considerable margin. However, similar to the comparisons of our method’s reconstruction performance, it should again be noted that the other approaches utilize different datasets and preprocessing steps. For example, Delaney et al. (2019) generated ECGs with multiple cardiac cycles, while Kuznetsov et al. (2021) focused on ECGs consisting of a single cardiac cycle. Furthermore, we find that the multi-domain VAE achieves a comparable MMD value as the ECG-only VAE. Comparing our method’s MMD to the gold standard MMD achieved on the same test dataset, we observe a 74% lower MMD value.

The population quality of the generated ED and ES anatomies is assessed by calculating population-wide cardiac anatomy metrics, which are commonly used in clinical practice, for both the 500 generated point clouds and the point clouds of the test dataset that we consider the gold standard for this analysis. Table 4 depicts the resulting values for the LV and RV volumes of each phase and the LV mass.

**TABLE 4:** Clinical metrics of meshed ED and ES anatomy point clouds generated by our method.

Phase	Clinical Metric	Gold Standard	Ours
ED	LV volume (ml)	141 (±30)	139 (±31)
	RV volume (ml)	170 (±34)	176 (±37)
ES	LV volume (ml)	59 (±15)	58 (±16)
	RV volume (ml)	78 (±20)	80 (±24)
ED/ES	LV mass (g)	102 (±28)	99 (±29)

Values represent mean (±standard deviation) in all cases

All clinical metrics show high degrees of similarity between generated and gold standard point cloud populations for both ED and ES phases, indicating that the VAE was able to successfully generate realistic virtual anatomies.

### 3.4 Combined Multi-Domain Generation

While our previous analyses have demonstrated the population quality of the generated ECGs and anatomies separately for each domain, we also want to investigate whether the same holds true for combined distributions of these outputs. To this end, we first calculate common clinical metrics combining ED and ES anatomies (LV SV (Eq. 10), RV SV (Eq. 10), LV EF (Eq. 11), RV EF (Eq. 11)) to assess mechanical cardiac function for both our generated and test dataset populations (Table 5).

**TABLE 5:** Clinical function metrics of meshed point clouds generated by our method.

Clinical Metric	Gold Standard	Ours
LV EF (%)	58 (±8)	57 (±9)
LV SV (ml)	82 (±21)	81 (±22)
RV EF (%)	55 (±7)	55 (±8)
RV SV (ml)	92 (±19)	96 (±22)

Values represent mean (±standard deviation) in all cases

We observe very good alignment between the clinical function metrics from all generated and gold standard meshed point clouds, indicating that our method is capable of synthesizing accurate ED-ES anatomy pairs.

Results presented up to this point demonstrate the ability of our method to produce realistic ECG populations, as well as ED and ES point clouds. In order to evaluate whether the method generates anatomy and electrocardiogram outputs preserving the correspondence between them, we select all cardiac anatomy and function metrics from Table 4 and 5 and concatenate them with the respective ECG signal to obtain a combined, low-dimensional representation of anatomy and ECG data for each case. We then calculate the MMD between the generated and test datasets consisting of the combined data representations for each case (Table 6). Similar to Table 3, we also determine the MMD between two random subsets of the test set as our gold standard value.

**TABLE 6:** Difference in randomly generated multi-modal distributions combining MRI-based anatomy and ECG-based electrophysiology.

Metric	Gold standard	Ours
MMD	5.02 × 10^–4^	4.72 × 10^–4^

Values represent mean in all cases

Our method obtains MMD values close to the gold standard ones, suggesting a good degree of coupling between the generated anatomy and ECG outputs.

### 3.5 Latent Space Analysis

**FIGURE 5:**
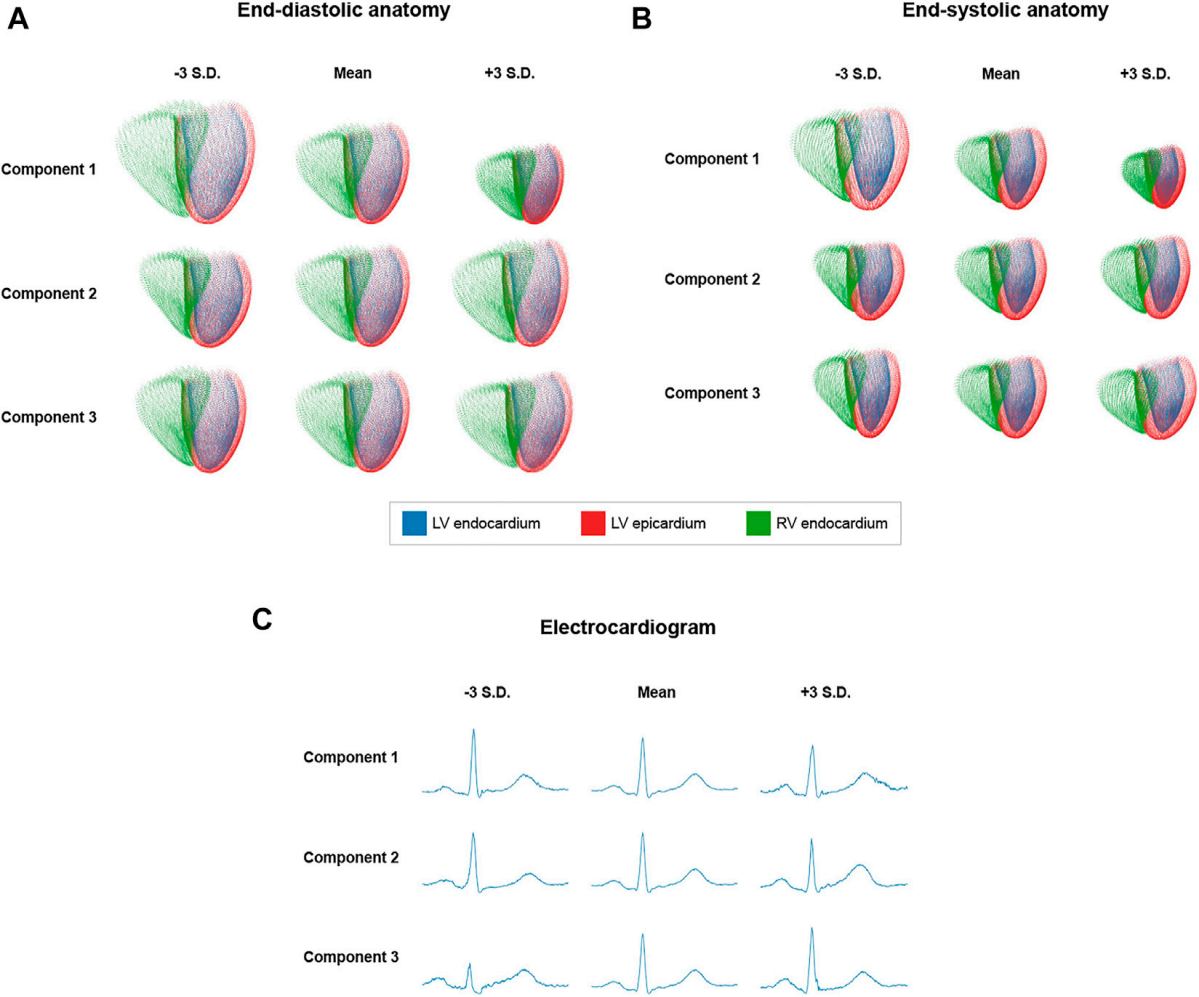
Effects of varying different latent space components by -3 standard deviations (S.D.) and +3 S.D. from their mean values on the generated ED **(A)**, ES **(B)**, and ECG **(C)** outputs.

A desirable feature of a variational autoencoder is the existence of an interpretable, disentangled latent space, in which different components are responsible for encoding various identifiable structural aspects of the generated output shapes. In order to analyze these characteristics for the proposed multi-domain VAE, we vary the values of each latent space dimension in both positive and negative directions while keeping the mean values for the remaining dimensions the same, and pass the resulting latent space vectors through the decoder to obtain outputs that correspond to the applied latent space changes. Three sample latent space components with easily visible effects on the generated multi-domain outputs are depicted in Figure 5.

Regarding the point cloud outputs, each component’s variation results in similar changes to the ED (Figure 5A) and ES (Figure 5B) anatomies, respectively. Component 1 controls the overall size of the point cloud and component 2 causes a tilt in the basal short-axis plane of the heart, while component 3 converts elongated, thin hearts to shorter and wider ones. Regarding the ECG data (Figure 5C), component 3 changes both the R-peak height and existence of the S-wave, component 2 increases the height of the P-wave and T-wave, while component 1 has an effect on the height and width of the R-wave as well as the height and sharpness of both the P and T-waves.

### 3.6 Cardiac Disease Classification

**FIGURE 6:**
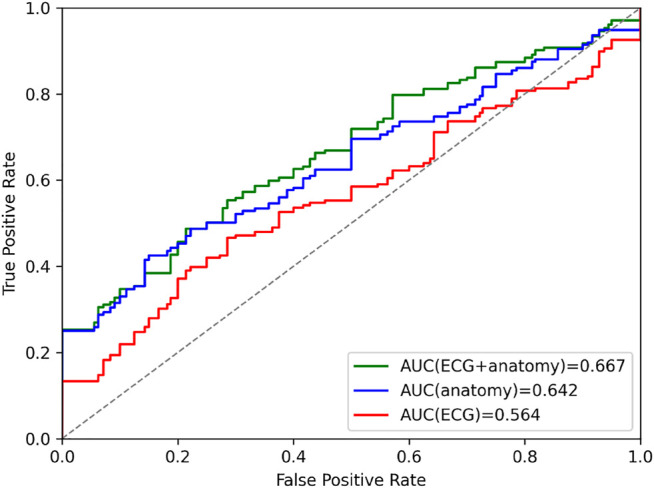
Area under curve (AUC) prediction differences in ROC curves of the cardiac disease classification results based on latent space representations of VAEs trained on combined anatomy and ECG, only anatomy, and only ECG data.

In order to further explore the VAE’s latent space, we investigate the utility of its compressed multi-domain representation of cardiac anatomy and physiology information for the task of cardiac disease classification, and compare its performance to similar single-domain representations. To this end, we first select the 150 healthy and 150 pathological United Kingdom Biobank cases described in Section 2.1 and use them as the basis for our binary disease classification task. We then pass the corresponding bitemporal anatomy and ECG data of each case through their respective encoders in the VAE to obtain the pertinent multi-domain latent space encodings, which serve as input features for the classification. Next, we repeat the same procedure using the point cloud encoders and the time-series encoder separately to calculate the single-domain encodings of bitemporal anatomy and electrophysiology information, respectively, for the same subjects. For each of the three resulting latent space datasets (multi-domain, anatomy-specific, and ECG-specific), we train a logistic regression classifier to identify subjects with cardiac disease. Figure 6 depicts the binary classification results of the 10-fold cross validation experiments for each combination of latent space datasets in the form of AUROC curves. We find that the multi-domain representation of anatomy and ECG achieves the highest AUROC score.

## 4 Discussion

In summary, we have demonstrated in our experimental results that the proposed multi-domain VAE can excel at a variety of different tasks despite the challenging multi-domain setting.

### 4.1 Reconstruction Accuracy

The point cloud branches are able to reconstruct complex 3D anatomical shapes with high accuracy on both a local and global level and for both the ED and ES phases of the cardiac cycle (Figure 3) with Chamfer Distances below the underlying image resolution (Table 2). This shows the high suitability of the anatomy-specific network architecture. In addition, it is able to accurately maintain class information identifying the different cardiac substructures and cope with anatomies at both the ED and the ES phase of the cardiac cycle. This indicates that an introduction of additional class information about important anatomical substructures or pathological areas (e.g. scar regions in the myocardium) or a further temporal extension is possible. In our experiments, we observe slightly higher distance values for the RV compared to the LV substructures and for the ED phase compared to the ES phase. We hypothesize that this is likely caused by the generally larger heart sizes that are represented by point clouds with the same resolution as the smaller hearts, which in and of itself leads to larger Chamfer Distance values. Therefore, we do not presume this to impede any future applications, as the differences are not due to any anatomical reasons. Furthermore, we find no erroneous overlappings of different anatomical substructures (e.g., at the interventricular septum) despite no loss term specifically enforcing such consistency. From these findings, we conclude that the point cloud branches are flexible and robust with respect to temporal and spatial variations and are able to capture the complexity of part-whole relationships in 3D structures, all of which are crucial for accurate cardiac anatomy modeling. These results are achieved despite the complex multi-domain setting in which separate point cloud branches for ED and ES as well as another ECG-based branch all share a single latent space and are trained jointly using a weighted combination of several loss function terms. This indicates that the good reconstruction performance of the point cloud-specific deep learning architecture is not limited to the single-domain setting, as in Beetz et al. (2021b), but can be applied effectively in conjunction with data from new domains while still maintaining the more challenging variational setup.

The time-series branches show a similarly good performance in the reconstruction task with a high degree of visual closeness between reconstructed and gold standard signals (Figure 3). We also find considerably lower RMSE and PRD scores than multiple benchmarks (Table 1). However, we interpret this finding as only an approximate comparison due to the usage of another dataset (MIT-BIH) and preprocessing steps to train and evaluate the benchmark methods. Nevertheless, while the MIT-BIH dataset has some differences compared to the United Kingdom Biobank dataset used in this work (e.g., ambulatory two-channel ECG vs. 12-lead ECG, 47 subjects vs. 1,300 subjects, multiple cardiac cycles vs. single cardiac cycle), they also share many similarities (e.g., both are ECG datasets with normal and pathological subjects, all compared methods focus on single lead ECG signals). In addition, we have applied similar filtering steps as the benchmark approaches (e.g., min-max normalization, same choice of sequence length) to the ECG signals in order to improve comparability between the two datasets. Hence, while a direct comparison with the other methods is limited by the dataset differences, the results still give an indication that the time-series branch architecture is able to successfully encode and decode different temporal patterns of lead II ECG signals. These findings are further corroborated by the similar reconstruction performance of the ECG-only VAE and the proposed multi-domain VAE. Since these results were achieved on the same United Kingdom Biobank dataset, they enable a direct comparison which is not affected by differences in the data or preprocessing steps between the methods. Hence, the similar RMSE and PRD values observed for both methods indicate that the multi-domain VAE was able to capture the ECG-specific information required for the reconstruction task similarly well as a single-domain ECG approach.

### 4.2 Generation of Virtual Multi-Domain Populations

In addition to the multi-domain VAE’s reconstruction ability, we also find it to be capable of generating arbitrarily-sized virtual populations of combined bitemporal anatomies and ECGs with a high degree of realism and correct levels of shape diversity. We are able to observe this visually in Figure 4, where typical shape changes in biventricular surfaces (e.g., overall size, basal plane tilt, ventricular thickness) and ECGs (e.g., R-peak height and width, P-wave peakedness, small noise levels in the signal) appear in the generated virtual examples in a similar way as in the real dataset.

The quantitative results further corroborate this finding in multiple ways. First, the generated ECGs from our VAE achieve lower MMD scores than the gold standard real ECGs from our test set (Table 3). On the one hand, the generally small values indicate that the distribution of real ECG signals is closely mimicked by the generated ones on both individual and population levels (Table 3). On the other hand, we hypothesize that the lower MMD scores for the generated ECGs are likely caused by the VAE’s ability to act as a regularizing self-prior and reduce noise. The proposed multi-domain VAE also achieves a comparable MMD score as the ECG-only VAE benchmark on the same United Kingdom Biobank dataset, which indicates that the ECG population was well captured despite the challenging inclusion of additional bitemporal anatomy information. Furthermore, our proposed method obtains lower MMD scores than multiple prior approaches in its ECG generation task. While this particular finding should again (similar to the reconstruction task) only be seen as an approximate comparison due to the usage of different datasets, it does nevertheless provide further evidence that the architectural design of the time-series branches can successfully convert random latent space samples into ECG populations. Second, the clinical volume-based metrics calculated for the population of generated anatomies closely resemble the ones obtained from the true gold standard test dataset, both in terms of their mean and standard deviation values (Table 4). This indicates that the point cloud branches are able to synthesize realistic biventricular shapes that accurately represent the morphological variety across the whole population. The network achieves this for both the ED and ES phases showing its architecture’s ability to function well with temporally related, but different shape distributions. Third, the clinical function metrics, which combine volume-based anatomical information from ED and ES phases, exhibit high degrees of similarity between the generated and gold standard anatomies in terms of both mean and standard deviation values (Table 5). This demonstrates that the synthesized anatomies do not only reflect a realistic population at ED or ES separately but also when considered as a combined bitemporal anatomy population. This correspondence between ED and ES shapes in the generated population is highly beneficial for multiple follow-up tasks (e.g. mechanical deformation modeling (Beetz et al., 2021c)). We also conclude from these results that the ED-ES correspondence information is likely captured in the shared latent space of the VAE and that the respective ED and ES point cloud branches are sufficiently powerful to correctly take into account cross-temporal information during training. Fourth, when combining ECG and bitemporal anatomy information in a unified representation, we find similar MMD values between synthesized and real gold standard populations (Table 6). This indicates that good correspondence is present not only between different cardiac phases but also between the generated ECG and anatomy data and that both the decoder branches and latent space information adequately model these inter-domain relationships. We note that while the selected cardiac metrics used to represent the anatomy in the unified representation only act as low-dimensional approximations of the full generated shapes for the MMD calculations, they were weighted accordingly to give the anatomical and ECG-based information a balanced influence in the combined representation. In general, since the aforementioned results were achieved using real ECG data, we hypothesize that the VAE could also be applied to synthetically-generated ECGs (e.g. via electrophysiology simulations based on mathematical models) for the task of generating personalized models of both normal and pathological data in real-time, which we wish to explore in detail in our future work.

### 4.3 Latent Space Quality

The positive results in the data generation tasks are likely significantly facilitated by the high quality of the latent space, which we observe to exhibit a good degree of disentanglement and interpretability (Figure 5). This can be seen by the clearly distinguishable effects that different individual latent space components have on the reconstructed anatomy and ECG outputs. We also find such latent space changes to cause gradual deformations of the output shapes of all domains while maintaining a realistic overall appearance even in case of larger deviations from the mean values. This indicates that the latent space learnt during training at least approximately resembles a multivariate normal distribution as enforced by the Kullback-Leibler divergence loss, as opposed to a more sparse and disordered representation that might lead to sudden unrealistic outlier shapes in the generated distributions. But compared to the zero mean parameterization of all Gaussians, we achieve better generation results when using the mean values predicted by the encoders on the training dataset to parameterize the latent space normal distributions for sampling. This shows that the actual latent space distribution still exhibits some differences to the target normal distribution. Nevertheless, this slight deviation is to be expected as the overall VAE loss represents a compromise between accurate reconstruction and latent space quality. We find the weighting parameter *β* to be crucially important for determining the optimal balance for the given dataset empirically, especially considering our highly challenging multi-domain setting. The similar shape changes observed in the ED and ES reconstructions corroborate the good choice of *β* further and demonstrate that the aforementioned high interpretabiliy of the latent space is retained even in the cross-domain case.

### 4.4 Cardiovascular Disease Classification

As evidenced by the cardiac disease classification results in Figure 6, the multi-domain latent space representation is able to successfully capture shape patterns related to both the healthy hearts and various cardiovascular pathologies in both the ECGs and the bitemporal anatomies. This offers the possibility to discover, visualize, and analyze pathology-specific feature combinations in both ECG and anatomy. One approach to achieve this would be to compare healthy and pathological latent spaces in terms of their respective mean representations or distributions and then reconstruct the corresponding anatomies and ECGs for each group to visualize the differences. Another possibility might be to identify the latent space components that are most predictive during the classification tasks and study what effects the corresponding changes in these latent components have on the reconstructed anatomies. Furthermore, when applying the multi-domain VAE trained on healthy subjects to diseased cases, we observe a slight decrease in reconstruction performance compared to unseen healthy cases, which also indicates that the network has learnt patterns specific to the healthy subpopulation. These results provide further proof of the importance of image-based and ECG shape analysis on both local and global scales for cardiac disease identification, which is in line with other previous findings (Mauger et al., 2019; Acero et al., 2022). One crucial difference to these prior works, however, is the combination of anatomy and ECG information in a compressed format that we observe to be more effective than similar approaches relying on either anatomy or ECG information alone (Figure 6). This multi-domain approach is particularly advantageous for the selected cardiovascular disease class containing different pathologies whose diagnoses are usually based on different modalities (e.g., cardiac MRI, ECG). Furthermore, we observe smaller differences in AUROC values between ECG + anatomy and anatomy-only information than between ECG + anatomy and ECG-only information. On the one hand, this might be due to the individual pathologies considered in the classification task that might be more easily predicted based on anatomy information. On the other hand, it could also be caused by our focus on only lead II ECG signals from a single heartbeat. This provides the ECG-only classifier with less information, which is in contrast to the high-dimensional multi-class 3D point cloud data that serves as input to the anatomy-based classifier. We also note that the latent space representation was obtained without any prior explicit training for the task of disease prediction, which outlines the potential for further improvements in directly finding pathology-specific compressed shape representation.

### 4.5 Architectural Design and Training

We have found the architectural design of our network (Figure 1C) to be highly suitable to process combined ECG and anatomy input data. The point cloud branch architectures (Figure 2A,B) are able to apply deep learning operations directly on point cloud data, which allows surface data of much higher resolution to be efficiently processed and used for storing anatomical shape information. This is in contrast to the widely-used voxelgrid representations (Çiçek et al., 2016; Bello et al., 2019; Xu et al., 2019), which are considerably less memory-efficient at managing surface-level data leading to lower resolution, longer processing times, and ultimately limit the overall accuracy of the modeled anatomy. Furthermore, each of the high-dimensional point clouds combines both the left and right ventricular anatomy and maintains separate labels for the LV endocardium, LV epicardium, and RV endocardium substructures. This results in a more holistic and accurate representation of the true 3D cardiac anatomy compared to the non-labelled single ventricle approaches and enables a more detailed and effective study of the structure-function interactions between MRI-based cardiac anatomy and ECG-based cardiac electrophysiology information.

As opposed to traditional shape modeling approaches, such as principal component analysis (Mauger et al., 2019; Acero et al., 2022), the deep learning architecture is able to capture significantly more complex and non-linear shape variations, which is important for the accurate modeling of the intricate interactions of both single-domain ECG and anatomy data, but especially in the multi-domain setting. In addition, no point-to-point correspondence is required in the point cloud dataset and no prior shape registration step needs to be applied, which makes the preprocessing steps considerably simpler, faster, and less error-prone compared to the PCA (Figure 1A,B). Another advantage of the VAE framework is its condensed latent space representation of the input data, which is useful for a variety of different tasks as shown in this work. The design of the time-series branches (Figure 2C,D) relies on a combination of convolutional, pooling, and fully connected layers, as opposed to recurrent layers such as Long Short-Term Memory (LSTM) or Gated Recurrent Units (GRU), and its good performance is in line with previous findings in ECG modeling (Zhu et al., 2019). For all branches, we hypothesize that the fully connected layers on both sides of the latent space in the encoder and decoder architectures provide the necessary power and flexibility to extract the relevant information for each domain from the shared latent space, while still accounting for inter-domain correspondence. Despite no specifically designed consistency loss between different output branches, we find that a careful empirical choice of weighting parameters in Eq. 3 and Eq. 5 in the domain-specific loss function components is sufficient to obtain high quality outputs both intra-domain and inter-domain. Finally, we note that the domain-specific data preprocessing of the proposed approach offers a certain degree of robustness and flexibility with regards to changes in the input data (e.g., different image resolutions in the cine MRI acquisitions), as both the 3D cardiac surface reconstruction and the ECG preprocessing steps can be adjusted as required in order to still be capable of creating 3D anatomy point clouds and ECG time series in a suitable format for the multi-domain VAE. For example, the same point cloud resolution can be maintained by the 3D surface reconstruction method despite changes in the underlying image resolution.

### 4.6 Limitations

The presented approach to multi-domain cardiac anatomy and physiology modeling also has some limitations. While it has previously been shown that the position and orientation of the heart with respect to the ECG electrodes on the torso significantly affects the ECG shapes (Mincholé et al., 2019), we did not include any torso information in this work. However, since the VAE was trained with paired anatomy and ECG information from real acquisitions, we hypothesize that the network is at least to some extent able to implicitly learn the effect of the torso on the output signals. We also note that since the 3D anatomy models were derived from 2D cine MRI acquisitions, any limitations (e.g., image resolution) or errors (e.g. slice misalignment due to inconsistent breath holds) introduced during the image acquisition or 3D reconstruction will affect the accuracy of the anatomical shapes. Similarly, we also note that the United Kingdom Biobank imaging study uses very established acquisition protocols and certain quality control measures that might not be fully representative of a standard clinical environment. While this makes the results easier to understand, it might also require some adjustments to the proposed methods in case of their application to different settings with a possibly larger variety of acquisition conditions and noise. While this study only focuses on lead II ECGs averaged across multiple cardiac cycles and thereby foregoes additional information from other leads and multi-heartbeat patterns, we believe that the core part of the architecture has the potential to be extended to the full-cycle 12-lead case. This could be achieved by first applying the same preprocessing steps to each of the 12 leads in order to represent each lead signal as a normalized 400-dimensional vector. The resulting vectors could then be concatenated and input into the ECG branch of the VAE. The ECG loss could be easily extended to include multiple leads by summing or averaging over the lead-specific mean squared errors. In addition, adjustments to the ECG branch architecture, training schedule, and possibly the lead-specific weighting terms in the loss function will likely be necessary to accommodate the increased difficulty of processing all 12 leads. Another limitation of the method is that no anatomical information about the atria is included in the model, which plays an important role in modeling electrophysiology. However, as the, to the best of our knowledge, first deep learning approach to combine anatomy and ECG data in a single data-driven model, we found the utilized information sources sufficient to demonstrate the feasibility and show the benefits of a multi-domain cardiac model. Information from other domains can be included into the model in future work, for example, as extra classes in the point cloud inputs, additional time series in the ECG inputs, or as new network branches altogether.

## 5 Conclusion

In this work, we have developed and evaluated a novel multi-domain VAE with the ability to capture combined cardiac anatomy and physiology information and their intricate interconnections in a single data-driven model. We have shown that the network can successfully handle the complex interdependencies of multi-domain datasets by reconstructing existing cardiac data from low-dimensional latent spaces with high accuracy and generating realistic populations of corresponding cardiac anatomies and ECGs. Furthermore, we have found an interpretable latent space in the VAE with each component responsible for a separate morphological change in anatomy and ECG outputs enabling a more localized analysis of cardiac health. Finally, we have observed that combined anatomy and ECG representations improve the identification of cardiovascular disease compared to single-domain approaches. This shows the utility and positive synergies of large-scale data integration from multiple sources in cardiology and opens up promising future research avenues for possible further multi-domain integration.

## Data Availability

The data analyzed in this study is subject to the following licenses/restrictions: The United Kingdom Biobank datasets used for network training and evaluation are not allowed to be shared publicly. Requests to access these datasets should be directed to https://www.ukbiobank.ac.uk. Generated virtual data can be made available upon reasonable request.
