# Supplementary material for: Multi-Domain Variational Autoencoders for Combined Modeling of MRI-Based Biventricular Anatomy and ECG-Based Cardiac Electrophysiology
Source: Front Physiol. 2022 Jun 8;13:886723. doi: 10.3389/fphys.2022.886723 (PMC9213788; doi:10.3389/fphys.2022.886723)
Supplement: Supplementary file 1 [file Table1.pdf]

Supplementary Material: Multi-Domain Variational Autoencoders for Combined Modeling of MRI-Based Biventricular Anatomy and ECG-Based Cardiac Electrophysiology

1 CARDIAC DISEASE DEFINITION

Table S1 depicts all cardiac diseases with their respective self-reported UK Biobank codes that were considered in the binary classification task.

Table S1. UK Biobank codes used to define "cardiac disease" in this work.

UK Biobank Code	Meaning
1066	Heart/cardiac problem
1074	Angina
1075	Heart attack/myocardial infarction
1076	Heart failure/pulmonary odema
1077	Heart arrhythmia
1471	Atrial fibrillation
1483	Atrial flutter
1484	Wolff Parkinson white/WPW syndrome
1485	Irregular heart beat
1486	Sick sinus syndrome
1487	SVT/supraventricular tachycardia
1078	Heart valve problem/heart murmur
1584	Mitral valve disease
1585	Mitral regurgitation/incompetence
1586	Aortic valve disease
1587	Aortic regurgitation/incompetence
1079	Cardiomyopathy
1588	Hypertrophic cardiomyopathy (HCM/HOCM)
1080	Pericardial problem
1589	Pericarditis
1590	Pericardial effusion
